# Molecular Crosstalk Between Flowering Time and Drought Adaptation in Cereal Crops

**DOI:** 10.3390/plants15132024

**Published:** 2026-06-30

**Authors:** Song Song, Xiaowei Fan, Nannan Zhang, Nan Lin, Guanfeng Wang

**Affiliations:** 1 College of Life Sciences, Henan Agricultural University, Zhengzhou 450002, China; 2 College of Agronomy and Biotechnology, China Agricultural University, Beijing 100193, China; 3 Sanya Institute of China Agricultural University, Sanya 572024, China

**Keywords:** drought, flowering time, cereal crops, photoperiod, phytohormone, sugar signaling

## Abstract

Increasingly frequent and severe drought events restrict global agricultural productivity. As sessile organisms, cereal crops have evolved phenotypic plasticity, drawing on drought escape (DE) and drought avoidance (DA) strategies to balance survival and reproduction. While the mechanisms governing photoperiodic flowering and drought responses are well characterized individually, their molecular intersection remains poorly understood. This review summarizes recent advances in the crosstalk between these two pathways. We highlight the divergent roles of core genetic hubs, such as florigen regulation, *GIGANTEA* (*GI*), DELLA proteins, and dual-function transcription factors (e.g., *ZmCCT*, *Ghd7*, *Ppd-H1*), and the breeding-selected alleles, including Green Revolution variants, that can partly uncouple stress tolerance from developmental penalties, though trade-offs often remain. Furthermore, we examine the internal networks driving this crosstalk, including circadian clock phase shifts, sugar and energy signaling through the trehalose-6-phosphate (T6P)-SNF1-related protein kinase 1 (SnRK1) module, and the antagonistic balance within phytohormone networks centered on abscisic acid (ABA). Finally, we propose that integrating epigenetic stress memory, systemic root-to-shoot signaling, and targeted CRISPR/Cas promoter engineering provides a useful conceptual framework for breeding climate-resilient, yield-stable crops.

## 1. Introduction

Increasingly frequent and severe droughts driven by climate change limit global agricultural productivity, particularly for major cereal crops [1,2]. As sessile organisms, plants depend on phenotypic plasticity and gene regulatory networks to survive adverse environments [3,4]. The timing of the floral transition is a major determinant of reproductive success and crop yield [5]. In cereal crops, this transition is regulated by the photoperiodic pathway, which monitors day length to align reproductive development with the season [6,7]. Drought not only causes cellular dehydration and stomatal closure but also directly interferes with these developmental programs [8]; it can disrupt circadian rhythms and photoperiodic perception, shifting flowering time [9].

Plants cope with drought mainly through two strategies: drought escape (DE) and drought avoidance (DA) [10,11]. DE is typically triggered by severe, continuous terminal drought, under which plants accelerate development to complete their life cycle before soil moisture is depleted [12,13]. Conversely, under mild-to-moderate or intermittent drought during early-to-mid stages, plants tend to adopt DA through physiological water conservation, which delays reproductive development to avoid the most moisture-sensitive periods and maintain survival [8,14]. Which strategy prevails depends on the timing, duration, and severity of the water deficit and on genotype-by-environment interactions, so the same regulatory hub can promote escape under one scenario and delay under another. Although adaptive in nature, these strategies often incur developmental penalties in agriculture, such as silking delays and an expanded anthesis-silking interval (ASI) in maize (*Zea mays*) [15].

The independent mechanisms governing plant responses to photoperiod and to drought have each been extensively studied. However, the intersection between these two pathways remains largely elusive [16,17]. Clarifying the crosstalk between photoperiodic flowering and drought adaptation is important for understanding how plants respond to complex environments [18], and it provides a basis for breeding yield-stable, climate-resilient crops [19,20]. This review first outlines the phenotypic plasticity and trade-offs of cereal crops under water deficit, then dissects the core molecular hubs that mediate the crosstalk between flowering and stress tolerance. We further describe the regulatory networks of circadian rhythms, sugar signaling, and phytohormone pathways through which environmental stress reshapes crop reproduction. Finally, we discuss genetic strategies to decouple stress tolerance from yield penalties via targeted genome editing.

## 2. Developmental Plasticity Under Drought Stress

Crop responses to water deficit are not fixed. They vary with genotype, with the intensity and duration of the stress, with the stage at which it strikes, and even between tissues of the same plant [3,14,21]. Plants classically resist drought in three ways that act at different levels of organization [10,11]. Drought escape (DE) is a developmental response: the plant accelerates flowering and grain set to finish its most sensitive stages before water runs short [10,13]. Drought avoidance (DA) is a whole-plant, water-conserving response—through stomatal closure, reduced leaf area, or deeper rooting—that protects tissue hydration but usually delays development [11]. Drought tolerance (DT) operates at the cellular level, sustaining metabolism at low water potential through osmotic adjustment and protective proteins; it can accompany either escape or avoidance rather than substitute for them [11]. Because the timing of flowering is set by developmental rather than cellular events, the crosstalk examined here runs mainly through DE and DA, with DT entering chiefly where it shares hormonal and sugar signals with the flowering pathway. The agronomic goal these strategies serve is reproductive resilience—the maintenance of fertility and grain yield during and after stress [3], which the balance struck between escape and avoidance ultimately determines.

### 2.1. Genotype and Stress Intensity-Dependent Responses

In major cereals, mild or intermittent drought during vegetative or early reproductive stages tends to trigger DA responses such as stomatal closure [8,22]. The accompanying drop in carbon assimilation delays the reproductive transition, protecting vulnerable floral organs from dehydration [11]. Severe terminal drought instead forces a DE strategy, accelerating development to ensure seed set before the soil is fully depleted [10,13].

**Figure 1:**
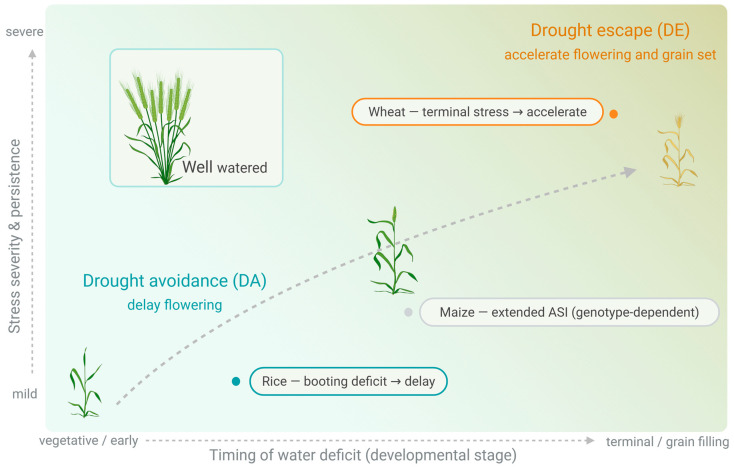
Drought escape and avoidance as a continuum set by the timing and severity of water deficit. Rather than a fixed dichotomy, the two strategies occupy regions of a landscape defined by the developmental stage at which deficit strikes (horizontal, vegetative or early to terminal or grain filling) and by stress severity and persistence (vertical, mild to severe). Mild or earlier deficit favors avoidance (lower left), where the plant delays the reproductive transition and stays vegetative; severe, terminal deficit favors escape (upper right), where it accelerates flowering and grain set to finish before water runs out, maturing and senescing early. The regions grade into each other through a broad, genotype-dependent overlap, and the dashed arrow shows that one genotype can move from avoidance toward escape as stress worsens. All marker positions and the schematic plants, including the boxed well-watered reference, are illustrative rather than quantitative. Among documented cases, a moderate deficit at booting delays heading in rice (avoidance), stress around flowering extends the maize anthesis-silking interval to a genotype-dependent degree (overlap), and terminal stress accelerates grain filling and senescence in wheat (escape).

This plasticity varies across different crop ecotypes. In rice (*Oryza sativa*), moderate water limitation during the booting stage frequently causes heading delay. However, terminal drought can specifically trigger a rapid escape response in upland or early-maturing varieties [17,23]. In wheat (*Triticum aestivum*), early drought delays development, whereas terminal stress accelerates grain filling and senescence [16]. In maize, the temporal desynchronization of male and female floral development is a typical stress-induced developmental arrest that extends anthesis-silking interval [24,25,26]. Because water deficit severely limits carbohydrate supply to the developing ear, the degree of ASI expansion varies among inbred lines, indicating that natural allelic variation strongly influences drought resilience. Beyond such differences between genotypes, a single genotype is itself flexible: the same plant can delay under one stress regime and accelerate under another, and its organs need not respond alike. These genotype- and context-dependent outcomes, which vary with both the severity and the timing of the water deficit, are summarized in Figure 1.

### 2.2. Evolutionary Trade-Offs and Yield Penalties

Drought-related developmental plasticity reflects an evolutionary trade-off between ecological adaptation and agricultural productivity [5,10]. Under either DE or DA, stressed plants prioritize survival and reproduction over maximizing biomass [27].

Although the DE strategy ensures reproductive success under terminal stress, the shortened vegetative phase limits canopy establishment [13]. This reduces biomass and the photosynthate allocated to sink organs, and thus yield [8,28]. The DA strategy also limits growth: prolonged stomatal closure curbs water loss but strongly inhibits photosynthetic carbon assimilation [29,30]. In farming, these protective mechanisms conflict with maximizing grain yield, underscoring the need to breed varieties that maintain yield under drought without developmental costs [19,20].

## 3. Core Molecular Hubs Integrating Flowering and Stress Responses

Drought-adaptive phenotypes in crops, such as shifts in flowering time, depend on molecular crosstalk among complex signaling pathways. This integration is controlled by genetic hubs that function in both environmental perception and developmental regulation. Florigen acts as the terminal signal for floral transition, and its transcriptional regulation under water deficit is a primary target of this crosstalk [31]. Although certain fundamental mechanisms are conserved, classic dicot models, specifically GIGANTEA (GI) and DELLA signaling, exhibit evolutionary divergence or genetic decoupling in cereal crops. In these crops, various dual-function transcription factors act to coordinate the trade-offs between stress tolerance and plant development.

### 3.1. Transcriptional Regulation of Florigens

As core molecular signals for the reproductive transition, florigens undergo complex transcriptional regulation under water deficit. Unlike the dicot model *Arabidopsis thaliana*, which mainly uses an abscisic acid (ABA)-dependent pathway to upregulate *FLOWERING LOCUS T* (*FT*) expression for drought escape [9,32], major cereal crops have evolved divergent regulatory networks to balance stress survival and reproductive development [13].

In rice, drought signals are integrated mainly by repressing the florigen pathway. During floral transition, water deficit downregulates the core florigen genes *Heading date 3a* (*Hd3a*) and *RICE FLOWERING LOCUS T1* (*RFT1*), as well as their upstream activator *Early heading date 1* (*Ehd1*) [33]. This transcriptional repression delays the floral transition and protects young panicles from irreversible dehydration damage, forming a central component of the DA strategy in rice, prioritizing maternal survival under extreme water deficit.

In maize, florigen signaling is similarly subjected to tight transcriptional control. Genome-wide association studies indicate that flowering time variation in temperate maize is cooperatively driven by the major florigen gene *ZEA CENTRORADIALIS 8* (*ZCN8*) and its paralog *ZCN12* [34]. Under drought conditions, the transcript levels of *ZCN8* in leaves decrease substantially, restricting the reproductive signals transported to the shoot apical and lateral meristems [35]. At the flowering stage, water deficit independently limits photosynthate flux to the developing ear, triggering ovary abortion and delayed silk growth that widen the anthesis-silking interval (ASI) [36].

Conversely, in temperate long-day crops like wheat that frequently encounter terminal drought, the DE mechanism predominates. In wheat, the PRR37-family photoperiod hub *Ppd-1* activates the florigen *TaFT1* under long days to promote heading [16]. Under drought, *TaFT1* is co-expressed with the stress-associated transcriptional repressor *TaDr1*, both showing increased expression in high-yielding cultivars [37]. This co-expression has been proposed to favour earlier flowering—consistent with a DE strategy that completes seed set before terminal drought—although the link currently rests on expression correlation rather than functional validation.

### 3.2. GIGANTEA in Cereals: Evolutionary Divergence and Knowledge Gaps

In *Arabidopsis*, the circadian clock component *GIGANTEA* (*GI*) acts as a central hub integrating photoperiodic signals with drought stress responses. Genetic studies demonstrate that *GI* is strictly required for the ABA-dependent DE response; *gi* mutants fail to accelerate flowering under water deficit [9]. At the molecular level, this integration relies on multiple intersecting pathways. GI positively regulates ABA biosynthesis by physically interacting with the ENHANCED EM LEVEL (EEL) transcription factor to promote *NCED3* expression [38]. Under drought stress, GI also facilitates the recruitment of CONSTANS (CO) to the *FT* promoter to activate transcription [32]. Additionally, the abundance of the GI protein is dynamically regulated by diurnal cycles and stress-induced targeted degradation, allowing for the continuous recalibration of photoperiodic sensitivity [39,40].

Major cereal crops exhibit evolutionary divergence in this integration pathway. Functional evidence for this divergence has been demonstrated in rice. While the rice ortholog *OsGI* is essential for maintaining normal photoperiodic flowering [41], physiological and transcriptomic analyses confirm that it is functionally uncoupled from the drought-induced reproductive delay, even though its own transcript levels shift under water deficit [33]. Specifically, drought-induced heading delay, the primary DA strategy in rice, remains fully functional in loss-of-function *osgi* mutants [33,42]; instead, the drought signal represses *Ehd1* and the downstream florigens *Hd3a* and *RFT1* independently of *OsGI*.

In the temperate cereals wheat and maize, the role of GI in the drought–flowering crosstalk remains unclear, largely because the available evidence is correlative rather than functional. Transcriptomic studies identify *GI* orthologs such as wheat *TaGI* as drought-responsive genes, though the direction of this response can vary with genotype and timing [43]. On its own, however, a shift in expression cannot show whether a gene transmits the drought signal or merely responds to it, and no loss- or gain-of-function study has yet tested this in wheat or maize. The pleiotropy of clock genes and the redundancy of polyploid genomes further complicate any single-gene interpretation; in hexaploid wheat, for instance, GI exists as three homoeologous copies. On present evidence, cereal *GI* orthologs are better regarded as downstream responders; establishing whether any of them act as genuine drought sensors will require knockout and overexpression lines assessed for flowering time under controlled drought, together with epistasis tests against the florigen and its upstream regulators. Addressing this gap is essential to avoid oversimplifying the translation of dicot models to complex cereal genomes.

### 3.3. Genetic Decoupling of DELLA and GA Signaling

In *Arabidopsis*, the balance between gibberellin (GA) and DELLA proteins links environmental stress to developmental transitions. Under optimal conditions, bioactive GA promotes the rapid degradation of DELLA repressors via the SCF-SLY1 proteasome pathway [44]. Drought stress disrupts this balance by transcriptionally downregulating GA biosynthesis [45]. The resulting GA deficiency stabilizes DELLA proteins and promotes their nuclear accumulation. Because they lack direct DNA-binding capability, DELLAs function through protein–protein interactions; they sequester and inhibit floral integrators such as CO and Phytochrome Interacting Factors (PIFs) to delay the floral transition [46,47]. DELLAs also enhance stress-responsive signaling pathways, reallocating metabolic resources toward survival [48]. In this dicot model, vegetative growth arrest is strictly coupled with reproductive delay.

In major cereal crops, DELLA signaling functions within a distinct agronomic context driven by “Green Revolution” alleles. Modern high-yielding wheat and rice varieties predominantly carry mutated alleles (e.g., wheat *Rht* and rice *sd1*) that lead to the constitutive stabilization of DELLA proteins or deficiency in GA synthesis. These mutations confer a semi-dwarf trait that improves lodging resistance and harvest index [49,50]. Despite the constitutive accumulation of these repressors, the severe floral delay observed in dicots does not occur in these cereals. Genetic profiling confirms that while DELLA stabilization restricts plant stature and activates stress-protective gene expression, it exerts minimal pleiotropic effects on flowering time, which remains independently governed by photoperiod and vernalization [51,52]. Thus, the growth-repressing and floral-repressing functions of DELLA are genetically uncoupled in cereals, allowing modern crops to maintain a lodging-resistant, high-yielding architecture without compromising reproductive phenology.

### 3.4. Dual-Function Transcription Factors

Plants have evolved specific molecular networks to integrate water deficit signals with the floral transition. Although stress- and development-related gene families are vast, transcription factors that simultaneously function as drought sensors and floral meristem determinants represent a highly specialized subset (Table 1). These regulators act across multiple pathways, conferring distinct adaptive strategies shaped by species and environment.

Under severe drought, certain crops activate dual-function repressors to execute a DA strategy, delaying flowering to prioritize vegetative survival. Within the ABA signaling network, the rice bZIP transcription factor gene *OsABF1* is highly induced by drought and indirectly represses the transcription of the floral activator *Ehd1*, thereby delaying reproductive development [23,53]. Similarly, OsbZIP23 directly binds and represses the floral organ identity gene *OsMADS14*, delaying flowering while enhancing dehydration tolerance [54]. Transcription factors also mediate hormonal crosstalk; for instance, rice *OsNAC2* strongly responds to ABA and upregulates repressors in the GA pathway, causing late flowering and dwarfism [55,56,57]. Unlike the dual-function repressors above, however, *OsNAC2* overexpression lowers drought and salt tolerance and reduces yield under drought, whereas its knockdown improves them, so here a flowering delay is decoupled from, rather than supportive of, stress survival. In maize, the CCT-family member *ZmCCT* is a well-characterized dual-function repressor. Molecular evidence confirms that *ZmCCT* acts as a transcriptional repressor, delaying flowering by directly repressing the clock and photoperiod genes *ZmPRR5* and *ZmCOL9*, while enhancing drought tolerance through direct targeting of stress-related genes such as *ZmHY5* and *ZmMPK3* [58]. Genetic analyses further indicate that natural variations at the *ZmCCT* locus enhance overall drought tolerance while delaying flowering [59]. However, this water-conserving strategy often incurs developmental penalties, such as the reduced biomass and severe reproductive arrest observed in wheat overexpressing *TaHDZipI-5* [60]. Such trade-offs are further exemplified by the rice CCT-family member *Ghd2*, which strongly delays heading but confers extreme drought sensitivity, highlighting the survival costs of prolonged vegetative development [61,62]. To balance this penalty, rice *OsWRKY11* exhibits dose-dependent regulation: low expression under mild drought promotes heading, whereas high expression under severe drought delays heading, allowing dynamic developmental adjustments based on stress severity [63].

Conversely, crops can also accelerate reproductive development under water restriction to execute a DE strategy. In rice, multiple pathways integrate drought signals to advance heading. The WOX family member *OsWOX13* acts as an upstream node, simultaneously upregulating drought-responsive factors (*OsDREB*) and floral pathway genes (*Hd3a* and *OsMADS14*), which enhances drought resistance while advancing the heading date [64]. Within the ABA-dependent pathway, the CCT-family member *Ghd7* exhibits functional plasticity: high expression under well-watered conditions enhances water retention, whereas negative feedback regulation by endogenous ABA under mild drought promotes earlier heading [65,66]. In an ABA-independent parallel pathway, the major quantitative trait locus (QTL) *Ghd7.1* (*OsPRR37*) responds to drought, accompanied by the differential expression of the MADS-box factor *OsMADS50* to accelerate the reproductive transition under water deficit [65].

**Figure 2:**
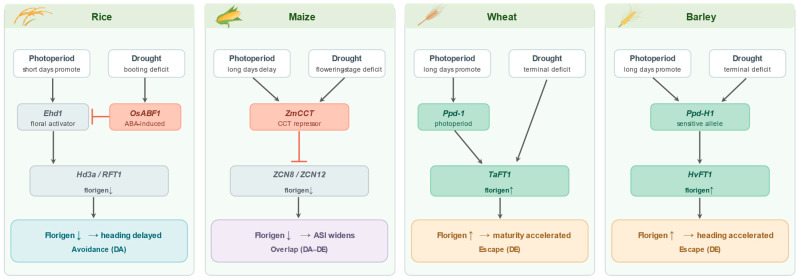
Florigen regulation under drought diverges across cereal crops. In each cereal, photoperiod and drought converge on the FT-type florigen, but water deficit drives it in opposite directions. In rice, drought-induced *OsABF1* indirectly represses the activator *Ehd1*, lowering *Hd3a*/*RFT1* and delaying heading (drought avoidance, DA); the response is dose-dependent, as mild deficit can instead promote heading. In maize, *ZmCCT* represses *ZCN8*/*ZCN12* and widens the anthesis–silking interval (ASI) to a genotype-dependent degree (DA–DE overlap). In wheat and barley, terminal drought instead raises florigen: long-day *Ppd-1* and water deficit both induce *TaFT1* in wheat, while in barley the sensitive *Ppd-H1* allele promotes early *HvFT1* expression and flowering, allowing escape and outyielding the insensitive allele under terminal drought (drought escape, DE). Pointed arrows, activation; bar-headed connectors, repression; node fill shows state (green, high or florigen-promoting; coral, active repressor; grey, low or repressed); ↑/↓, florigen up-/downregulation; outcome-bar color, strategy (teal, DA; amber, DE; lavender, overlap). Interactions shown are documented in the respective cereal.

Decoupling drought tolerance from late-flowering yield penalties is a primary objective of molecular breeding [67]. Certain transcriptional hubs demonstrate the potential to uncouple stress responses from developmental arrest. For example, in maize, overexpression of the Nuclear Factor Y (NF-Y) subunit *ZmNF-YB2* increases field water-use efficiency while stabilizing flowering time and final grain yield under severe dehydration [68]. The efficacy of these targets depends not only on single-gene functions but also on allelic variation and genotype-by-environment (G×E) interactions. For instance, in barley (*Hordeum vulgare*) the photoperiod hub *Ppd-H1*, an ortholog of rice *OsPRR37*, induces the florigen *HvFT1* under long days [69]. Its sensitive allele drives drought escape under severe terminal drought, yielding better than insensitive alleles in specific environments [70]. Characterizing these dual-role regulators provides a basis for decoupling abiotic stress tolerance from developmental penalties in crop improvement. The divergent photoperiod–drought integration and the resulting DA/DE outcomes across the four cereals are summarized in Figure 2.

**Table 1:** Dual-function transcription factors regulating both drought stress responses and flowering time in cereal crops.

Gene Family	Gene Name	Species	Evidence & System	Causality	Core Mechanism	Ref.
bZIP	*OsABF1*	Rice	Functional: OE, biochemical	Y	Strongly induced by drought; indirectly represses the floral activator *Ehd1* to delay reproductive development and avoid stress.	[23,53]
bZIP	*OsbZIP23*	Rice	Functional: KO, OE, biochemical	Y	Directly binds and represses the floral organ identity gene *OsMADS14*, delaying flowering to enhance dehydration tolerance.	[54]
NAC	*OsNAC2*	Rice	Functional: OE, KD	Y	Responds to ABA and up-regulates GA pathway repressors, leading to late flowering and dwarfism.	[55,56,57]
WRKY	*OsWRKY11*	Rice	Functional: OE (dosage), biochemical	Y	Dosage-dependent dual effect: under mild drought induces *OsMADS14/15* to promote heading; under severe drought represses *Ehd1* via an OsWRKY11–Hd1–DTH8 complex to delay it.	[63]
WOX	*OsWOX13*	Rice	Functional: OE, KO	Y	Mediates stress response and early flowering by up-regulating *OsDREB1A/1F* and *Hd3a/OsMADS14* to facilitate a drought escape strategy.	[64]
CCT	*Ghd2*	Rice	Functional: OE, genetic, biochemical	Y	Delays flowering by up-regulating the repressor *OsCO3*; confers drought sensitivity by activating senescence-associated genes via interaction with 14-3-3 and OsARID3.	[61,62]
CCT	*Ghd7*	Rice	Functional (flowering); Association: expression (drought)	Partial	Down-regulated by endogenous ABA under mild drought, thereby relieving its repression of the floral transition to promote earlier heading.	[65,66]
CCT/PRR	*Ghd7.1 (OsPRR37)*	Rice	Functional (flowering); Association: expression (drought)	Partial	Up-regulated in the ABA-independent drought-escape pathway, together with *OsMADS50*; the link rests on expression changes rather than functional validation.	[65]
MADS-box	*OsMADS50*	Rice	Association: expression	N	Synergizes with *Ghd7.1* in an ABA-independent network to advance heading under water deficit.	[65]
MYB-related	*OsCCA1*	Rice	Functional: OE, genetic	Y	Core clock hub; integrates endogenous ABA signals and circadian rhythms to co-regulate heading date and dehydration tolerance.	[71]
MYB-related	*ZmCCA1*	Maize	Association: expression (maize); Heterologous (*Arabidopsis*)	Partial	Core clock component; in maize only splice-variant expression responds to drought, while delayed flowering and drought tolerance have been shown only in transgenic *Arabidopsis*.	[72,73]
NF-Y	*ZmNF-YB2*	Maize	Functional: OE, field	Y	Stabilizes flowering time and maintains grain yield under severe dehydration without severe developmental penalties.	[68]
NF-Y	*ZmNF-YA3/7*	Maize	Functional: CRISPR-KO, biochemical	Y	Activates *ZmFT-like12* to promote flowering; binds *bHLH92*, *FAMA*, and *MYC4* to enhance ABA/JA-mediated stress resistance.	[74]
CCT	*ZmCCT*	Maize	Functional: OE, biochemical; Association: QTL	Y	Natural alleles affect comprehensive drought tolerance; acts as a core photoperiod determinant to modulate flowering.	[58,59]
Zn-Finger	*ZmDi19-5*	Maize	Functional: genetic, biochemical	Y	Activates floral repressor *ZmCOL3* and represses stress negative regulator ZmHsf08; its activity is modulated by ZmFKF1b.	[75]
HD-Zip	*TaHDZipI-5*	Wheat	Functional: OE	Y	Confers dehydration tolerance but incurs severe penalties, including extreme delayed flowering and reduced biomass.	[60]
NAC	*TaSNAC8-6A*	Wheat	Functional: OE, ChIP-seq; Association: GWAS	Y	Acts through the *TaABF*–ABRE module to enhance lateral-root development and water-use efficiency under drought; under water limitation it slightly delays flowering while raising grain yield.	[76]
PRR	*Ppd-H1*	Barley	Functional: NIL; Association: field	Y	Core photoperiod hub and drought-signal integrator. The photoperiod-sensitive allele flowers early to escape terminal drought and buffers drought-induced down-regulation of *HvFT1*; non-functional alleles show stronger repression and delayed development.	[69,70]

Notes. **Evidence & system**. Evidence classes (Functional, Biochemical, Association, Heterologous) are combined with the method used. Functional, gene-level evidence from direct manipulation, overexpression (OE), knockout (KO), knockdown (KD), CRISPR editing, or near-isogenic/introgression lines (NIL); biochemical, direct DNA-binding or protein–protein interaction assays (EMSA, ChIP-seq/DAP-seq, Y1H/Y2H, BiFC); Association, correlation inferred from expression or transcriptomic data, or from GWAS/QTL mapping; Heterologous, function shown only in another species (e.g., *Arabidopsis*); field, multi-environment field trials; genetic, classical genetic/allelic analysis; dosage, expression-level-dependent effect. **Causality** (experimental validation of the drought–flowering link). Y, gene-specific functional evidence under drought; Partial, the gene’s flowering or drought role is functionally validated, but drought-responsiveness of the link rests on expression data or pathway inference; N, the link is supported only by expression correlation or genetic mapping.

## 4. Endogenous Signaling Networks

**Figure 3:**
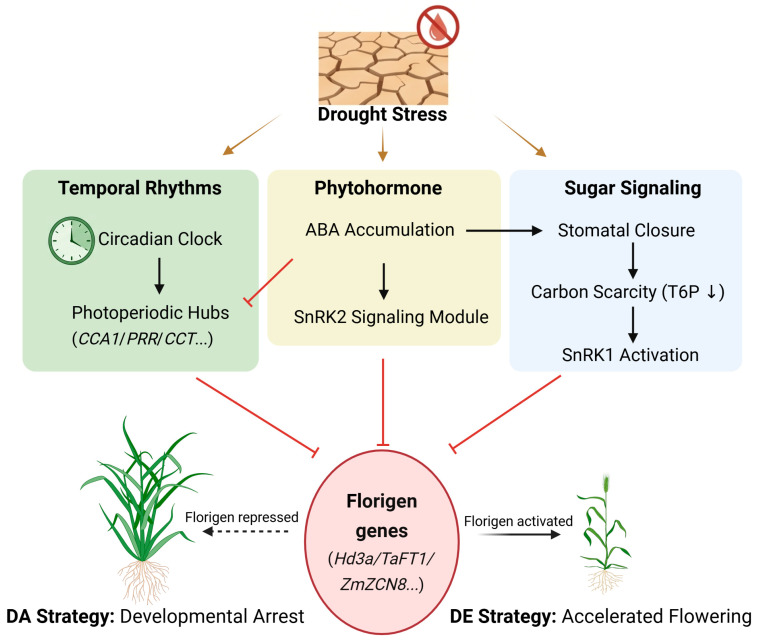
Regulatory networks coordinating drought response and reproductive transitions in cereal crops. Drought stress signals are integrated via three primary modules: temporal rhythms, phytohormones, and sugar signaling. ABA acts as a central mediator, activating the SnRK2 signaling module while simultaneously repressing photoperiodic hubs and inducing stomatal closure. This closure restricts carbon assimilation, leading to carbon scarcity, which triggers decreased T6P levels and subsequent SnRK1 activation. These upstream pathways converge to dictate the expression of core florigen genes (e.g., *Hd3a*, *TaFT1*, *ZmZCN8*). The robust repression of florigen signals (dashed arrow) arrests floral development, driving the drought avoidance (DA) strategy. Conversely, the activation of florigen (solid arrow) accelerates the reproductive transition, facilitating the drought escape (DE) strategy. Throughout, pointed arrows denote activation or sequential progression and red bar-headed connectors denote repression. The colored panels group the three modules.

The reproductive transition of crops under drought stress requires the integration of multiple internal pathways. The circadian clock and photoperiodic networks establish the temporal rhythm of the stress response, defining the timing for developmental shifts. Furthermore, photosynthetic limitations under drought trigger internal carbon scarcity. Sugar signaling molecules monitor energy reserves to regulate reproductive progression and arrest development when energy is severely deficient. These temporal and metabolic cues are subsequently transduced through hormone networks centered on ABA. Interactions among different phytohormones then trigger specific phenotypes such as shifts in flowering time or organ abortion. Together, temporal rhythms, carbon metabolism, and hormonal signaling dictate the phenotypic transition between DA and DE strategies (Figure 3).

### 4.1. Temporal Control via the Circadian Clock

The plant circadian clock synchronizes internal physiological rhythms with external environmental fluctuations via transcription-translation feedback loops comprising components such as *CIRCADIAN CLOCK ASSOCIATED 1* (*CCA1*) and *pseudo-response regulators* (*PRRs*) [77,78,79]. Under water deficit, the circadian system acts as a hub linking environmental information to developmental decisions. By adjusting its rhythms through phase resetting and sensitizing stress responses via circadian gating, the clock regulates the switch between DA and DE strategies [80,81]. This temporal fine-tuning enables crops to balance defense responses and reproductive development.

Drought-induced osmotic signals first act on the clock’s input pathways, triggering phase shifts by perturbing core clock components. In barley, the photoperiodic hub gene *Ppd-H1*, which encodes the core clock component *HvPRR37*, integrates this signal. Under water deficit, the florigen *HvFT1* is down-regulated and floral development slows, with the extent of this penalty depending heavily on the *Ppd-H1* allele. Lines carrying a functional, photoperiod-sensitive allele buffer development strongly and resume growth quickly once water returns, whereas lines with a non-responsive allele suffer a marked delay [69]. Similarly, in rice, drought alters the rhythmic expression of core elements like *OsTOC1* [65]. These clock shifts propagate to the downstream photoperiodic network to drive divergent developmental choices between DA and DE.

Under the DA strategy, the transcriptional repressor *OsPRR73* accumulates under osmotic stress, reinforcing its inhibition of the florigen activator gene *Ehd1* to delay heading [82,83]. *OsCCA1* also activates ABA signaling components like *OsbZIP46* to maintain stomatal regulation and water homeostasis, supporting the developmental pause [71]. Similarly, the maize ortholog *ZmCCA1* integrates drought signals via alternative splicing; its floral-repressor activity has been shown in transgenic *Arabidopsis*, whereas in maize the evidence is expression-based [72,73]. In contrast, under the DE strategy, the photoperiodic network relieves developmental constraints. Rice utilizes two parallel drought escape pathways that are either dependent on or independent of ABA [65]. In the ABA-dependent pathway, drought-induced ABA accumulation represses the expression of the core negative regulator *Ghd7*, relieving its inhibition of downstream florigen genes (*Hd3a* and *RFT1*) to advance heading. Meanwhile, in the ABA-independent network, drought downregulates the transcription of *OsPRR37*, accompanied by the differential expression of regulatory genes such as *OsMADS50* to promote an earlier reproductive transition [65].

Despite belonging to the same PRR family, *OsPRR37* and *OsPRR73* exhibit opposing expression patterns and functions in drought response: *OsPRR37* represses *Ehd1* under normal conditions but is transcriptionally downregulated under drought to relieve this inhibition and promote early flowering; whereas *OsPRR73* is stress-induced to reinforce *Ehd1* inhibition and delay flowering [65,82]. Beyond transcriptional remodeling, the photoperiodic network also establishes rapid response mechanisms through protein–protein interactions [84]. For instance, in maize, the photoreceptor protein ZmFKF1b physically binds and sequesters the drought stress protein ZmDi19-5, buffering excessive repression of the flowering program [75]. Additionally, the dual-function transcription factor ZmNF-YB2, functionally associated with the clock network, maintains photosynthetic homeostasis while modulating flowering time under drought [68].

### 4.2. Energy Metabolism and Sugar Signaling

While the circadian network sets the timing of the drought response, the state of energy metabolism strongly influences whether reproductive development can be sustained. Stomatal closure under water deficit restricts photosynthetic carbon assimilation and drives intracellular carbon starvation [85]. Beyond serving as metabolic substrates, sugars act as signals in their own right. Sucrose reports carbon status systemically through the phloem and glucose is sensed locally by hexokinase, while trehalose-6-phosphate (T6P) tracks cellular sucrose and sets the activity of the energy sensor SNF1-related protein kinase 1 (SnRK1) [86,87]. When sucrose, and therefore T6P, is abundant, SnRK1 is held inactive and biosynthesis proceeds; when carbon assimilation falls, T6P declines and SnRK1 is released [88,89,90], repressing the growth-promoting target of rapamycin (TOR) kinase and arresting anabolic development [91]. Because T6P reports sucrose status through this single kinase relay, its developmental consequences depend on where the signal is read and which targets are locally available. A developing sink can therefore be arrested under severe deficit, while under milder deficit the floral program is left free to proceed.

**Figure 4:**
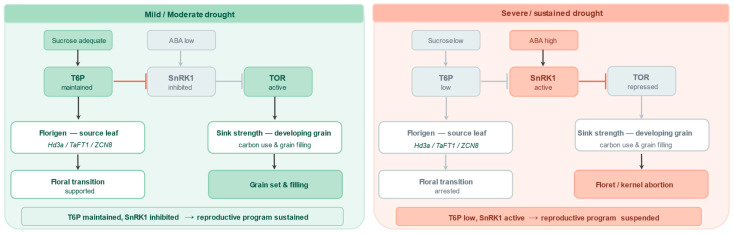
The T6P–SnRK1–TOR module gates reproductive output under contrasting drought intensity. The module sets two outputs, florigen in the source leaf and sink strength in the developing grain. Under mild to moderate drought (**left**), carbon is adequate, T6P keeps SnRK1 inhibited and TOR active, florigen supports the floral transition, TOR sustains grain sink strength, and the reproductive program is sustained. Under severe or sustained drought (**right**), carbon falls, T6P drops, and SnRK1 is released and further activated by rising ABA, repressing TOR; florigen and grain sink strength both decline, the floral transition is arrested, florets and kernels abort, and the reproductive program is suspended. Node color marks state (green, active or promoting; coral, active under stress or inhibiting; grey, low or off; white, pathway nodes not assigned a state). Pointed arrows denote activation or progression and bar-headed connectors denote repression; their color marks whether each link is operative in that regime (colored, operative; grey, inactive).The florigen genes *Hd3a*, *TaFT1* and *ZCN8* (rice, wheat and maize) stand in for *Arabidopsis FT*. The grain branch is consistent with cereal evidence of floret abortion in wheat and invertase-linked kernel abortion in maize, whereas the florigen branch and the activation of SnRK1 by ABA are extrapolated from *Arabidopsis*.

This context dependence is clearest in the contrast between source and sink tissues. In a mature leaf, the principal source organ, T6P reflects the sucrose available for export and feeds the photoperiodic pathway upstream of florigen; in the dicot model it is required for the floral transition and for *FT* expression in the leaf, coupling photosynthetic carbon status to the systemic floral signal [88]. What the leaf reads out is close to a binary commitment, whether to license a reproductive program. A developing grain or floret behaves differently. As a sink, its T6P reflects imported sucrose and sets sink strength, the capacity to draw and use carbon, through local control of sucrose unloading and utilization [90,92]. Here the output is graded, a continuous adjustment of how forcefully the sink competes for assimilates. The sensing step is shared, with SnRK1 responding to the local T6P level, but the targets available in a photosynthesizing leaf and an importing grain differ, so the same signal becomes a flowering decision in one tissue and a partitioning decision in the other (Figure 4). The sink arm of this logic has been shown directly in maize. Expressing the rice trehalose-6-phosphate phosphatase gene *OsTPP1* under a floral promoter in the developing ear and its female florets lowered T6P in the ear spikelets, raised their sucrose content, and increased kernel set and yield under both well-watered and drought conditions. The same enzyme expressed in ovules was instead detrimental to yield, indicating that only particular cells use T6P as a control point on sink strength [93].

Under severe drought the depletion of photoassimilates drives T6P low enough to activate SnRK1 in source tissues, which shifts assimilate partitioning away from reproductive organs. Developing inflorescences, anthers, and ovules are the dominant carbon sinks in cereals and are acutely sensitive to this withdrawal of supply [36,94]. Activated SnRK1 represses TOR and reprograms organ development [91]; in wheat the result is arrested spike growth and floret abortion [95]. In maize, drought suppresses cell wall and vacuolar invertase activity in the ear, so sucrose is not hydrolyzed and the hexose-to-sucrose ratio that supports cell division collapses, weakening kernel sink strength, intensifying competition among kernels, and causing apical kernel abortion [96]. This carbon-sensing failure is the metabolic core of the DA strategy, in which the ongoing reproductive program is suspended to preserve vegetative survival.

Under mild drought the same module produces the opposite developmental outcome. Carbon assimilation falls, but T6P does not drop far enough to fully release SnRK1, so sugar signaling is maintained and the DE program proceeds [88]. In barley, the two mature strands of a single miR172b precursor move in opposite directions under drought, yet both favor drought escape. When miR172b-5p drops, the trehalose-6-phosphate synthase (TPS) transcript it normally cleaves is spared, raising trehalose and sustaining T6P signaling, while the rising miR172b-3p cleaves the mRNAs of AP2-like floral repressors to bring flowering forward [97]. Because the precursor itself is not induced by drought, these opposite responses must be post-transcriptional, most likely arising from strand selection that favors one arm over the other.

The activation state of SnRK1 is a central determinant of reproductive fate under drought, and beyond carbon status it is also linked to ABA signaling. In *Arabidopsis*, the protein phosphatase 2C (PP2C)-SNF1-related protein kinase 2 (SnRK2) module that constitutes the core of ABA signaling has also been proposed to keep SnRK1 inactive. When ABA is low, SnRK1 is held within PP2C-SnRK2 complexes, an association that requires the PP2C. ABA then triggers the disassembly of these complexes, releasing and activating SnRK1 [98]. This core module is conserved in land plants, but whether it also regulates SnRK1 in cereals, for example through their SAPK kinases, remains untested. Should it do so, ABA would restrain reproductive growth not only by closing stomata and limiting carbon supply but also by activating SnRK1, reinforcing the shift from DE toward DA.

Source and sink responses of this kind are averages over anatomically complex organs, and single-cell and spatial transcriptomic methods now resolve them to the level of individual cell types. Within a water-stressed leaf the response is far from uniform. Single-cell analysis shows mesophyll cells, the main site of assimilation, halting energy-intensive metabolism while the epidermis activates defense genes, and spatial profiling resolves two mesophyll populations under mild drought, one near the leaf margins and the vasculature enriched for ABA-dependent stress transcripts and a more distal population running an iron-starvation program [99]. The floral transition is itself a meristem-level decision, and the same approaches are beginning to resolve the distinct cell states of meristematic and vascular tissue [99]. Locating where T6P falls, where SnRK1 becomes active, and where the floral signal is produced within this cellular geography is the resolution at which source and sink behavior will ultimately be understood.

### 4.3. Phytohormone Networks

In *Arabidopsis*, the hormonal network translates environmental stress into developmental decisions through precise signal transduction [100]. As the primary stress phytohormone, ABA modulates flowering time through the *SnRK2-ABA-responsive element-binding factor* (*AREB*)*/ABF* signaling module, which directly targets floral integrators such as *SUPPRESSOR OF OVEREXPRESSION OF CO 1* (*SOC1*) and *FT* [32,101]. Its core components act in both directions. The ABA INSENSITIVE 2 (ABI2) phosphatase dephosphorylates ABA INSENSITIVE 5 (ABI5) and lowers its stability, weakening ABI5-driven activation of the floral repressor *FLOWERING LOCUS C (FLC)* and thereby promoting flowering [102], whereas the SnRK2-ABI5-FLC module conveys the repressive effect of ABA on flowering [103,104]. This circuitry is, however, a dicot paradigm. Cereals do not regulate flowering through an *FLC*-type repressor; the closest *FLC* relatives in rice act as floral activators rather than repressors, and in the temperate cereals the vernalization response runs through the distinct *VERNALIZATION (VRN)* loci [52].

In cereal crops, the phenotypic output of ABA-regulated flowering depends strongly on the severity and duration of the water deficit. Intracellular ABA signals are primarily transduced by the SnRK2 family kinases (designated as stress/ABA-activated protein kinases, SAPKs, in rice) [105], which function as molecular switches to relay stress severity to distinct developmental targets. Under mild or short-term drought triggering a DE strategy, lower levels of ABA accumulation can act through the activation of specific floral promotion pathways. In rice, for instance, the ABA-activated SAPK10 kinase can promote the formation of the florigen activation complex (FAC) by phosphorylating the OsFD1 protein, demonstrating the potential to accelerate heading [106]. However, under severe or prolonged drought inducing a DA strategy, high ABA accumulation exerts an inhibitory effect. Once activated, the SAPK8 kinase phosphorylates OsABF1 to enhance its transcriptional activity. This phosphorylated OsABF1 directly represses the core floral activators *Ehd1* and *Ehd2*, and recruits the Polycomb Repressive Complex 2 (PRC2) complex to maintain long-term epigenetic silencing at these loci [107].

Furthermore, hormonal responses in maize and wheat more often involve desynchronized floral development. In maize, drought-induced ABA accumulation exerts an asymmetric inhibitory effect on male and female inflorescences, specifically arresting ear development and silk elongation. This effect results in a widened anthesis-silking interval, a hallmark of the DA strategy [108]. In wheat, ABA catabolism and homeostasis are closely linked to reproductive tolerance. Disruption of ABA homeostasis under drought suppresses early spike differentiation. This developmental delay is often accompanied by the abortion of spikelets or florets, leading to a mismatch between spike development and the vegetative growth cycle [109].

The reproductive outcome ultimately turns on the balance between ABA and the growth-promoting hormones, and the severity of the stress is what sets that balance. Under mild deficit, ABA stays low enough that GA and cytokinin signaling continue to support growth and the floral transition, sustaining the DE strategy; as the deficit deepens, rising ABA suppresses both pathways and tips the balance toward the DA strategy and developmental arrest. Within the GA module, breeding has partly uncoupled the DELLA repressors from flowering, yet severe drought overrides this. Under drought, ABA-activated OsSAPK9 degrades OsNAC120, a hub that activates GA biosynthesis and represses ABA biosynthesis, and SLR1 further inhibits it, so GA falls and ABA rises, braking growth in favour of survival [110]. Cytokinin sits at the other node of the balance, sustaining the sink strength of reproductive organs by maintaining invertase activity. Under severe drought, rising ABA weakens cytokinin signaling, leaving the developing florets at a disadvantage in the competition for assimilate [111].

Auxin ties this hormonal network to the wider stress response. It acts as an integrator that couples growth and development to environmental cues, working through stress-responsive gene expression, crosstalk with ABA, and the remodeling of root architecture under water deficit [112]. In cereals this integration reaches flowering-time control directly. In maize, the flowering-time regulator ZmCCT drives endogenous auxin signaling and interacts with ZmAux/IAA8 and the E3 ubiquitin ligase ZmWIPF2, a complex that activates downstream drought-defense genes and improves root growth and water status under deficit [113]. Across these pathways, ABA-centered signaling interweaves with GA, cytokinin, and auxin to set the reproductive fate of a cereal crop under drought, acting at once on floral gene expression, on the pace of development, and on the source-sink relationships that supply the grain.

## 5. Conclusions and Future Perspectives

In conclusion, cereal crops have evolved sophisticated regulatory networks to coordinate drought responses with photoperiod-induced flowering programs. One major axis of this crosstalk is the tight coupling between SnRK2 kinase-mediated stress sensing and florigen-centered developmental regulation, which operates alongside the circadian, sugar-signaling, hormonal, and source–sink modules discussed above. Through the flexible modulation of key hubs such as *ZmCCT*, *Ghd7*, and *Ppd-H1*, crops adopt either DE or DA strategies to balance survival and reproduction under water deficit. Although the molecular framework is increasingly clear, understanding how crops adapt to complex natural environments requires research in several directions.

Elucidating long-distance signaling from root sensors to the shoot apical meristem (SAM) is essential for understanding whole-plant coordinated responses. Although osmotic stress at the barley root is known to alter the expression of shoot circadian clock genes [81], the specific molecular carriers that relay this information remain largely unknown in cereals. Studies in dicot models provide a starting point. The *Arabidopsis* root-derived peptide CLE25 moves systemically to activate leaf BAM receptors and promote ABA accumulation [114]; hydraulic signals driven by xylem tension provide rapid physical feedback of water status [115]; and phloem-mobile small RNAs (e.g., *miR156/172*) participate in systemic developmental regulation [116]. Which of these candidates carries drought information to the cereal SAM, and whether the same carrier operates across rice, wheat, barley, and maize or the four have diverged, remains unknown. Resolving it will require tracing the signal from its source to its destination. Reciprocal grafting between stressed and unstressed genotypes can establish whether a drought cue is root-derived and graft-transmissible; sap profiling of the xylem and phloem would identify the peptides, small RNAs, and metabolites moving under water deficit; labeled peptides can be traced as they travel between organs; and meristem-specific reporter lines would reveal where the signal is received and which cells at the SAM respond. Applied side by side across the four cereals, these approaches would separate a conserved root-to-shoot module from species-specific wiring and connect root stress perception to the floral transition.

Integrating stress memory into the drought-flowering crosstalk remains a priority for future research. Dehydration during early vegetative growth often has a lasting influence on later flowering decisions through epigenetic modifications. This transcriptional memory depends on the persistent enrichment of H3K4me3 and the stalling of RNA polymerase II at specific genomic loci [117,118]. This memory operates against a backdrop of fast, reversible signaling that resets each time water returns, so the chromatin marks bias the eventual flowering decision rather than determine it. The marks are also short-lived, decaying within days to weeks and largely erased at each generation, which keeps their influence on the cereal floral transition limited and, for now, hard to quantify [118]. Future studies will need to move beyond single-gene validation to resolve how global epigenetic remodeling alters the chromatin landscape of key regulatory hubs and how these imprints are transmitted to the SAM during cell division. Uncovering this epigenetic crosstalk, which turns early physiological stress into late developmental change, will clarify the stability of crop adaptive strategies.

The key challenge in translating these mechanisms into yield is precisely decoupling stress tolerance from developmental penalties. In both natural evolution and domestication, drought resistance is often tightly linked with alterations in flowering timing, a genetic trade-off that hinders high-yield breeding. Transgenic studies have demonstrated the feasibility of breaking this linkage; for instance, overexpressing the nuclear factor *ZmNF-YB2* in maize enhances water retention under drought while maintaining normal silking dates and ear development, resulting in significant yield gains without penalties under well-watered conditions [68]. Future molecular design breeding should utilize CRISPR/Cas technologies to precisely engineer cis-regulatory elements within the promoters of key hubs (e.g., *Ghd7* or *ZmCCT*), specifically targeting motifs that respond to water deficit (such as ABA-responsive elements, ABREs) without disrupting the original photoperiodic sensing network [119,120]. Several constraints temper this prospect. Hubs of this kind are pleiotropic, so an edit that retunes the drought response can also shift grain number and plant height, and its effect is strongly genotype-by-environment dependent, helpful under one photoperiod or water regime and neutral or costly under another. The difficulty compounds in hexaploid wheat, where three homeologs of each hub must be edited together against a large and redundant genome, a harder proposition than in diploid rice or maize. A realistic validation path would move stepwise, editing a single candidate motif such as an ABRE in the *Ghd7* or *ZmCCT* promoter, confirming at the transcript level that drought-responsive promoter activity is reduced, retuned, or made more specific while normal photoperiodic expression is preserved, then phenotyping the lines across water regimes and field environments with heading date, the anthesis-silking interval, and grain yield as readouts, and verifying homeolog coverage explicitly in wheat. Such fine-tuned promoter engineering offers a promising strategy for developing climate-resilient cultivars that achieve high yields without sacrificing developmental stability.

## Data Availability

Data are contained within the article.

## Abbreviations

The following abbreviations are used in this manuscript:

ABA	Abscisic acid
ABF	ABRE-binding factor
ABI2	ABA INSENSITIVE 2
ABI5	ABA INSENSITIVE 5
ABRE	ABA-responsive element
AREB	ABA-responsive element-binding factor
ASI	Anthesis-silking interval
BiFC	Bimolecular fluorescence complementation
CCA1	CIRCADIAN CLOCK ASSOCIATED 1
ChIP-seq	Chromatin immunoprecipitation sequencing
CO	CONSTANS
CRISPR	Clustered regularly interspaced short palindromic repeats
DA	Drought avoidance
DAP-seq	DNA affinity purification sequencing
DE	Drought escape
DT	Drought tolerance
Ehd1	Early heading date 1
EMSA	Electrophoretic mobility shift assay
FAC	Florigen activation complex
FLC	FLOWERING LOCUS C
FT	FLOWERING LOCUS T
GA	Gibberellin
GI	GIGANTEA
GWAS	Genome-wide association study
G×E	Genotype-by-environment
Hd3a	Heading date 3a
KD	Knockdown
KO	Knockout
NF-Y	Nuclear Factor Y
NIL	Near-isogenic line
OE	Overexpression
PP2C	Protein phosphatase 2C
PRC2	Polycomb Repressive Complex 2
PRR	Pseudo-response regulator
QTL	Quantitative trait locus
RFT1	RICE FLOWERING LOCUS T1
SAM	Shoot apical meristem
SAPK	Stress/ABA-activated protein kinase
SnRK1	SNF1-related protein kinase 1
SnRK2	SNF1-related protein kinase 2
SOC1	SUPPRESSOR OF OVEREXPRESSION OF CO 1
T6P	Trehalose-6-phosphate
TOR	Target of rapamycin
TPS	Trehalose-6-phosphate synthase
Y1H	Yeast one-hybrid
Y2H	Yeast two-hybrid
